# The initial effectiveness of liposomal amphotericin B (AmBisome) and miltefosine combination for treatment of visceral leishmaniasis in HIV co-infected patients in Ethiopia: A retrospective cohort study

**DOI:** 10.1371/journal.pntd.0006527

**Published:** 2018-05-25

**Authors:** Charles Abongomera, Ermias Diro, Alan de Lima Pereira, Jozefien Buyze, Kolja Stille, Fareed Ahmed, Johan van Griensven, Koert Ritmeijer

**Affiliations:** 1 Médecins Sans Frontières, Abdurafi, Ethiopia; 2 Department of Clinical Sciences, Institute of Tropical Medicine, Antwerp, Belgium; 3 Department of Internal Medicine, University of Gondar, Gondar, Ethiopia; 4 Médecins Sans Frontières, Addis Ababa, Ethiopia; 5 Public Health Department, Médecins Sans Frontières, Amsterdam, The Netherlands; Saudi Ministry of Health, SAUDI ARABIA

## Abstract

**Background:**

North-west Ethiopia faces the highest burden world-wide of visceral leishmaniasis (VL) and HIV co-infection. VL-HIV co-infected patients have higher (initial) parasitological failure and relapse rates than HIV-negative VL patients. Whereas secondary prophylaxis reduces the relapse rate, parasitological failure rates remain high with the available antileishmanial drugs, especially when administered as monotherapy. We aimed to determine the initial effectiveness (parasitologically-confirmed cure) of a combination of liposomal amphotericin B (AmBisome) and miltefosine for treatment of VL in HIV co-infected patients.

**Methodology/Principal findings:**

We conducted a retrospective cohort study at a Médecins Sans Frontières—supported health center in north-west Ethiopia. We included VL-HIV co-infected adults, treated for VL between January 2011 and August 2014, with AmBisome infusion (30 mg/kg total dose) and miltefosine orally for 28 days (100 mg/day). Proportions of initial treatment outcome categories were calculated. Predictors of initial parasitological failure and of death were determined using multivariable logistic regression. Of the 173 patients included, 170 (98.3%) were male and the median age was 32 years. The proportion of patients with primary VL (48.0%) and relapse VL (52.0%) were similar. The majority had advanced HIV disease (n = 111; 73.5%) and were on antiretroviral therapy prior to VL diagnosis (n = 106; 64.2%). Initial cure rate was 83.8% (95% confidence interval [CI], 77.6–88.6); death rate 12.7% (95% CI, 8.5–18.5) and parasitological failure rate 3.5% (95% CI, 1.6–7.4). Tuberculosis co-infection at VL diagnosis was predictive of parasitological failure (adjusted odds ratio (aOR), 8.14; p = 0.02). Predictors of death were age >40 years (aOR, 5.10; p = 0.009), hemoglobin ≤6.5 g/dL (aOR, 5.20; p = 0.002) and primary VL (aOR, 8.33; p = 0.001).

**Conclusions/Significance:**

Initial parasitological failure rates were very low with AmBisome and miltefosine combination therapy. This regimen seems a suitable treatment option. Knowledge of predictors of poor outcome may facilitate better management. These findings remain to be confirmed in clinical trials.

## Author summary

North-west Ethiopia faces the highest burden world-wide of visceral leishmaniasis (VL) and HIV co-infection. VL treatment outcomes in HIV co-infected patients are associated with high initial treatment (parasitological) failure and recurrence rates after cure (relapse). With secondary chemoprophylaxis, the risk of relapse can be reduced. However, with the current VL treatment regimens, the initial parasitological failure rates remain high. In this study, we aimed to determine the initial effectiveness of a combination of liposomal amphotericin B (AmBisome) and miltefosine for treatment of VL in HIV patients in Ethiopia. We conducted a retrospective study using routine program data from a Médecins Sans Frontières—supported health center in north-west Ethiopia. We included 173 adult VL-HIV co-infected patients treated for VL with a combination of AmBisome and miltefosine. Initial cure rate was 83.8%, death rate 12.7% and parasitological failure rate 3.5%. Tuberculosis co-infection at VL diagnosis was predictive of initial parasitological failure. Predictors of death were age >40 years, hemoglobin ≤6.5 g/dL and primary VL. Initial parasitological failure rates were very low with AmBisome and miltefosine combination therapy. This regimen seems a suitable treatment option. Knowledge of predictors of poor outcome may facilitate better management. These findings remain to be confirmed in other studies.

## Introduction

Visceral leishmaniasis (VL) is a protozoan infection caused by the *Leishmania donovani* species complex [1]. In East Africa and the Indian subcontinent, it is caused by *L*. *donovani*, whereas in the Mediterranean region and South America, by *L*. *infantum* [2]. Ethiopia is among the top six high burden countries, with approximately 3.2 million people at risk and 3400–5000 VL cases occurring annually [3–5]. North-west Ethiopia faces the highest burden world-wide of VL-HIV co-infection, an estimated 20% of VL patients are HIV co-infected [6]. HIV infection influences the clinical course of VL: it reactivates latent *Leishmania* infection, increases VL severity, and negatively affects treatment outcomes [7]. VL in turn promotes the progression of HIV infection [7].

As to VL treatment outcomes, both higher (initial) parasitological failure rates and higher relapse rates have been described [7,8]. To reduce the relapse rate, secondary prophylaxis is the way to go [7,9–11]. We have recently documented the effectiveness, safety and feasibility of pentamidine secondary prophylaxis—started after parasitological cure was achieved—in Ethiopian VL-HIV co-infected adults [9,11]. However, achieving parasitological cure remains challenging, as co-infected patients have shown poor treatment response to all available antileishmanial drugs especially when administered as monotherapy [7].

Several studies have shown that pentavalent antimonials cause severe adverse events (cardiotoxicity, nephrotoxicity, hepatotoxicity, pancreatitis) resulting in high case fatality rates [7,12–18]. Antimonials have also been shown to stimulate HIV-1 replication *in vitro* [19]. In East Africa, high case fatality rates of 6.8% to 33.3% have been reported [14–16]. Due to the high case fatality rates, the World Health Organization (WHO) recommends that pentavalent antimonials should ideally not be used as a first line treatment for VL in HIV co-infected patients [20].

In comparison to antimonials, there is relatively limited clinical experience with miltefosine—a newer antileishmanial agent [7,15,20,21]. An Ethiopian study comparing miltefosine and the antimonial—sodium stibogluconate (SSG), showed that miltefosine was safer (lower death rates: 1.6% *vs*. 6.8%) but had lower initial effectiveness (higher initial parasitological failure rates: 17.5% *vs*. 2.3%) [15]. These high parasitological failure rates increase the potential for the emergence of resistant parasites [21]. Because patients with parasitological failure are potential reservoirs of resistant parasites, they are a major public health concern, especially in East Africa where the main mode of transmission of *Leishmania* parasites is anthroponotic [21]. Furthermore, miltefosine has a long half-life of approximately one week, and can develop resistance with a single point mutation [22]. The most optimal way to use miltefosine would be in combination with another antilesihmanial agent [21,23].

Several studies from the *L*. *infantum* areas of the Mediterranean region, in small numbers of co-infected patients, showed that liposomal amphotericin B was safe and effective [7,20]. Based on these findings and absence of similar studies from other VL endemic areas, the WHO recommended liposomal amphotericin B as the first line treatment for VL in HIV co-infected patients [20]. However, liposomal amphotericin B (AmBisome) monotherapy at a total dose of 30 mg/kg also had limited effectiveness in Ethiopia, with initial parasitological failure rates of 32.8% [24].

Combination treatment has been used in tuberculosis, HIV and malaria with good outcome and is increasingly being used for VL [23,25]. We reasoned that, as both AmBisome and miltefosine had been found to be safe, but with high initial parasitological failures as monotherapy, the combination of the two drugs with different modes of action and non-overlapping toxicity might yield a safe regimen able to decrease the high initial parasitological failure rates [7,15,20,21,24,26]. In 2011, Médecins Sans Frontières (MSF) introduced a compassionate treatment regimen of AmBisome and miltefosine combination as first line treatment for VL in HIV co-infected patients. In this study, we aimed to determine the initial effectiveness (cure, death and parasitological failure rates) of this regimen for treatment of VL in HIV co-infected patients in Ethiopia.

## Methods

### Study setting

The study was conducted at Abdurafi health center—an MSF supported health facility located in a remote town in Amhara region, in northwestern Ethiopia. The MSF support focuses on clinical management of VL, HIV and concomitant infections. It is a major VL treatment site in Ethiopia and medical services are free of charge. The majority (>95%) of VL patients treated at the health center, are young adult males working on the large-scale agricultural schemes in the northwestern lowlands.

### Study design and population

We conducted a retrospective cohort study using routine program data. In the main (per-protocol) analysis, we included all VL-HIV co-infected patients diagnosed between January 2011 and August 2014, aged ≥18 years, treated with an initial VL treatment regimen composed of a combination of AmBisome and miltefosine. Patients that discontinued treatment, defaulted, were transferred-out or had a missing VL treatment outcome were excluded.

We also conducted a sensitivity analysis that is similar to an intention to treat analysis. In this analysis, we also included patients that defaulted or were transferred-out, and considering that they probably had *Leishmania* parasites at exit, they were all classified as having parasitological failure.

### VL diagnosis

Patients with prolonged fever, splenomegaly and wasting were considered VL suspects and underwent further diagnostic evaluations [20]. Patients without prior VL treatment history (primary VL) were first screened using the rK39 rapid diagnostic test (IT-Leish, Bio-Rad laboratories, USA)[27] and a positive result confirmed VL. Those testing negative were screened with the leishmania direct agglutination test (DAT, Royal Tropical Institute, Amsterdam, The Netherlands)[28] and a high titer (≥1:3200) confirmed VL. Those with an intermediate DAT titer (1:800–1:1600) underwent tissue aspiration (spleen, bone marrow or lymph node) and a positive result confirmed VL. Patients with prior VL treatment history (relapse VL) underwent tissue aspiration and a positive result confirmed VL. A clinical diagnosis was made in patients [primary VL (with negative rK39 test results and intermediate DAT results) and relapse VL] who were contra-indicated for spleen aspirate (i.e. spleen size ≤2 cm, bleeding tendency, pregnant, severely anemic, jaundiced or in a state of collapse) or who declined a bone marrow aspirate and didn’t have palpable lymph nodes. Furthermore, a clinical diagnosis was also made in patients [primary VL (with negative rK39 test results and intermediate DAT results) and relapse VL] with negative bone marrow aspirate results but persistent strong VL clinical suspicion in the absence of differential diagnoses [24,29].

### HIV diagnosis and treatment

HIV positive status was defined by two positive results of serological tests performed in parallel {KHB (Shanghai Kehua Bio-engineering Co-Ltd, Shanghai, China) and STAT-PAK (Chembio HIV1/2, Medford, New York, USA)} and confirmed by the ELISA test {ImmunoComb (Orgenics ImmunoComb II, HIV 1&2 Combfirm)}. Antiretroviral therapy (ART) prescription was according to national guidelines and tenofovir, lamivudine and efavirenz combination was the most common first line regimen [30].

### VL treatment

The initial treatment regimen was a combination of liposomal amphotericin B (AmBisome, Gilead Sciences) at a total dose of 30 mg/kg, divided into 6 infusions of 5 mg/kg on alternate days and miltefosine (Impavido, Paladin Labs, Montreal, Canada) administered orally for 28 days (100 mg/day). Patients that showed a slow treatment response received a second course of the combination regimen at the same dosage (treatment extension). Slow treatment response was defined as a substantial parasite reduction after finishing the treatment course (day 29) as compared to baseline (parasite decrease ≥2 log-grades but parasitology result was still positive) [29,31]. Patients that showed no parasitological response to the combination treatment received sodium stibogluconate (Albert David Ltd., Kolkata) at a dose of 20 mg/kg/day by intramuscular injection for a minimum of 30 days (rescue therapy). No parasitological response was defined as no substantial parasite reduction at the end-of-treatment as compared to baseline (parasite decrease ≤1 log-grade and parasitology result was still positive) [29,31].

### VL treatment outcomes

Our main outcome of interest was the initial treatment outcome which was defined as the treatment outcome after completion of the first VL treatment course. The categories of initial treatment outcome included: cure, death, parasitological failure, defaulter and transfer-out. Parasitological tests were performed at the end-of-treatment in all co-infected patients except for those without palpable spleen or lymph nodes and who refused bone marrow aspirate, or for those with a contraindication for spleen aspirate. For this category of patients, cure was assessed clinically. Patients with parasitological failure received additional treatment (retreatment) and the subsequent treatment outcome was classified as retreatment outcome. Treatment outcome at discharge was either the initial treatment outcome or where applicable the retreatment outcome. The categories of treatment outcome at discharge were as for the initial treatment outcome.

Cure was defined as improvement in symptoms and signs of VL after treatment initiation (i.e. absence of fever, decrease in spleen size, increase in hemoglobin, weight gain) and a negative parasitological test at the end-of-treatment. Parasitological failure was defined as a positive parasitological test at the end-of-treatment. Death from all causes during VL treatment at the health center were documented. Defaulting was defined as absconding from treatment. Transfer-out was defined as referral to another hospital facility. Treatment discontinuation was defined as discontinuation of a VL treatment regimen prior to using less than 90% of the total recommended dosage.

### Data collection and measurement of variables

Since the program onset, clinical data were collected using standardized data collection tools and stored in electronic databases. The databases were updated on a daily basis by data managers. The data were collected at admission through history taking, clinical examination, laboratory and/or radiological investigations, and treatment prescriptions (VL and ART regimens). The following variables were assessed from patient history: age (years), sex, residential status [migrant worker (an individual who seasonally relocates to another area in search of work); settler (an individual who has been relocated to another area by the state) and resident (an individual who has permanently lived within a specific area for a duration of 2 or more years)], duration of illness (months) and VL treatment history (primary, relapse).

The following variables were assessed by clinical examination: weight (kilograms), height (meters)/length (centimeters), body mass index [BMI; weight in kilograms ÷ (height in meters)^2^], spleen size (centimeters), the level of weakness, ascites, peripheral edema, bleeding and jaundice. The spleen size (centimeters) was measured from the junction of the anterior axillary line and the left coastal margin to the tip of the spleen. Weakness severity was defined according to MSF guidelines [29] as follows: [State of collapse: unable to sit up unaided and cannot drink unaided; severely weak: cannot walk 5 meters without assistance; other types of weakness were classified as “other”].

The following variables were assessed by laboratory and/or radiological investigations. The mode of diagnosis of HIV was defined above (see HIV diagnosis). Using a microscope with a 10X eyepiece and 100X oil objective, tissue parasite grading were as follows: [0 (0 parasites/1000 fields); 1+ (1–10 parasites/1000 fields); 2+ (1–10 parasites/100 fields); 3+ (1–10 parasites/10 fields); 4+ (1–10 parasites/field); 5+ (10–100 parasites/field); 6+ (>100 parasites/field)]) [31]. Hemoglobin level was measured using a hematology analyzer—Beckman Coulter A^c^T diff, Beckman Coulter Inc., 2003, USA. CD4 count was measured at baseline and every six months after ART initiation using the FACS counter (BD FACS Calibur flow cytometer, 2009, USA). Tuberculosis diagnosis and WHO clinical staging were according to WHO guidelines [32,33].

### Statistical methods

The primary outcome was the initial treatment outcome (cure, death or parasitological failure). The proportion of individuals with the different outcome categories (excluding patients that defaulted or were transferred-out), were calculated with 95% Wilson confidence intervals (CI). In secondary analysis, the association of initial treatment outcome with VL treatment history was assessed by the Chi-squared or Fisher’s exact test.

Predictors of initial parasitological failure and predictors of death were determined. Predictors of parasitological failure were analyzed among patients with parasitological failure or cure, whereas predictors of death were analyzed among patients who died or stayed alive (cured or parasitological failure). The choice of variables analyzed as predictors was based on literature review and consideration of variables available in the dataset. To overcome the problem of substantial missing baseline CD4 count results, we created a composite marker for advanced HIV disease, defined as having either additional WHO stage IV disease [33] or a CD4 count <50 cells/μL at VL diagnosis [34]. Continuous variables were categorized based on information from literature and a recent study on predictors of death [35]. The association between predictors and parasitological failure or death were first assessed with Chi-squared or Fisher’s exact test. When the *p*-value was <0.1, the predictor was included in a multivariable logistic regression model. Non-significant variables (*p*-value ≥0.05) were removed step by step until no more variables could be dropped.

Lastly, a sensitivity analysis similar to an intention to treat analysis was performed. Defaulters and transfer-outs were included in this sensitivity analysis; and considering that they probably had *Leishmania* parasites at exit, they were all classified as having parasitological failure. All the statistical methods described above were then repeated. All statistical analyses were performed with Stata version 14.

### Ethics approval

Ethics approval was received from the Institutional Review Board of the Institute of Tropical Medicine, Antwerp, Belgium, and the Ethical Review Committee of the Institute of Public Health, Gondar University, Ethiopia. This research fulfilled the exemption criteria set by the MSF Ethical Review Board (ERB) for a posteriori analyses of routinely collected clinical data, and thus did not require MSF ERB review. It was conducted with permission from the Medical Director of the MSF Operational Centre Amsterdam.

## Results

**Fig 1:**
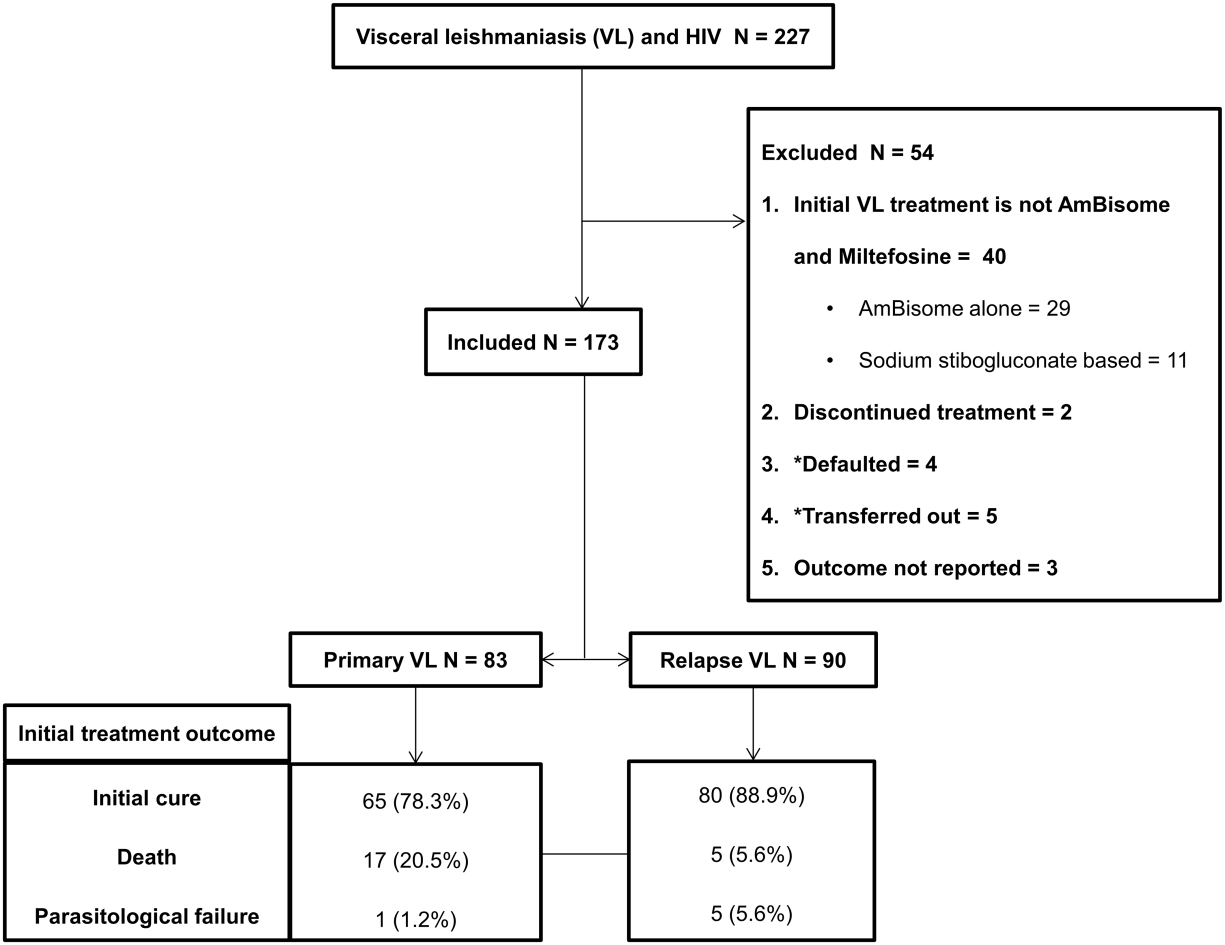
Flow diagram showing the number of patients in the main (per-protocol) analysis and their outcomes. * Defaulters and transfer-outs were included in the sensitivity analysis.

Between January 2011 and August 2014, 227 patients were diagnosed with VL-HIV co-infection and treated at the Abdurafi health center. Forty patients were treated with other VL treatment regimens (AmBisome alone, n = 29; SSG based, n = 11). Two patients were started on AmBisome and miltefosine combination treatment, however miltefosine was later discontinued. The reason for discontinuing miltefosine was because of miltefosine stock-out rather than adverse event. Both of these patients completed AmBisome monotherapy and one was cured and the other died. Four patients defaulted, 5 were transferred out and 3 had unknown treatment outcome. These 54 (23.8%) patients were excluded from the main (per-protocol) analysis. A total of 173 patients were included in the main analysis (Fig 1).

A comparison of characteristics of patients excluded or included in the study is shown in S1 Table.

### Demographic and clinical characteristics

Most patients were male (n = 170; 98.3%), residents (n = 101; 59.1%) and young (median age of 32 years; interquartile range [IQR] 28–39). The proportion of patients with primary VL (n = 83; 48.0%) and relapse VL (n = 90; 52.0%) were similar. Most patients had advanced HIV disease (n = 111; 73.5%) and were on ART prior to VL diagnosis (n = 106; 64.2%) (Table 1).

**Table 1:** Demographic and clinical characteristics of visceral leishmaniasis and HIV co-infected patients treated with a combination of liposomal amphotericin B (AmBisome) and miltefosine by MSF in Ethiopia from January 2011 to August 2014, by visceral leishmaniasis treatment history.

Characteristic	Total (N = 173)	Primary VL (N = 83)	Relapse VL (N = 90)	*P*
**Sex, n (%)**				
- Male	170 (98.3%)	80 (96.4%)	90 (100.0%)	0.11^a^
- Female	3 (1.7%)	3 (3.6%)	0 (0.0%)	
**Age (years), median (IQR)**	32.0 (28.0–39.0)	32.0 (27.0–37.0)	34.0 (28.0–40.0)	0.07^b^
**Age (years), n (%)**				
- 18–40	141 (81.5%)	73 (88.0%)	68 (75.6%)	0.04^c^
- >40	32 (18.5%)	10 (12.1%)	22 (24.4%)	
**Residential status, n (%); n = 171**				
- Migrant worker	70 (41.0%)	34 (41.5%)	36 (40.5%)	0.89^c^
- Resident	101 (59.1%)	48 (58.5%)	53 (59.6%)	
**Spleen size (cm), median (IQR); n = 170**	6.0 (4.0–10.0)	6.0 (3.0–10.0)	6.0 (4.0–10.0)	0.62^b^
**Spleen size ≥11 cm, n (%)**				
- Yes	28 (16.5%)	13 (15.7%)	15 (17.2%)	0.78^c^
- No	142 (83.5%)	70 (84.3%)	72 (82.8%)	
**Hemoglobin level (g/dL), median (IQR); n = 168**	8.2 (6.7–9.2)	8.1 (6.5–9.1)	8.3 (7.1–9.8)	0.11^b^
**Hemoglobin level ≤6.5 g/dL, n (%)**				
- Yes	40 (23.8%)	21 (25.6%)	19 (22.1%)	0.59^c^
- No	128 (76.2%)	61 (74.4%)	67 (77.9%)	
**Body mass index (kg/m**^**2**^**), median (IQR); n = 164**	16 (15–18)	16 (15–18)	17 (15–18)	0.39^b^
**Body mass index** <16 **Kg/m**^**2**^, **n (%)**				
- Yes	68 (41.5%)	36 (46.8%)	32 (36.8%)	0.20^c^
- No	96 (58.5%)	41 (53.3%)	55 (63.2%)	
**Tuberculosis, n (%); n = 170**				
- Yes	39 (22.9%)	16 (19.8%)	23 (25.8%)	0.35^c^
- No	131 (77.1%)	65 (80.3%)	66 (74.2%)	
**Jaundice, n (%); n = 169**				
- Yes	5 (3.0%)	3 (3.7%)	2 (2.3%)	0.67^a^
- No	164 (97.0%)	78 (96.3%)	86 (97.7%)	
**Duration of illness (months), median (IQR); n = 164**	1.0 (1.0–2.0)	1.0 (1.0–2.0)	1.0 (1.0–2.0)	0.06^b^
**Duration of illness ≥2 months, n (%)**				
- Yes	60 (36.6%)	36 (46.2%)	24 (27.9%)	0.02^c^
- No	104 (63.4%)	42 (53.9%)	62 (72.1%)	
**Bleeding, n (%); n = 167**				
- Yes	5 (3.0%)	2 (2.5%)	3 (3.5%)	1.00^a^
- No	162 (97.0%)	79 (97.5%)	83 (96.5%)	
**Weakness, n (%)**^d^; **n = 170**				
- Collapse	1 (0.6%)	0 (0.0%)	1 (1.1%)	0.29^a^
- Severe	26 (15.3%)	15 (18.5%)	11 (12.4%)	
- Other	143 (84.1%)	66 (81.5%)	77 (86.5%)	
**Edema and/or ascites, n (%); n = 168**				
- Yes	13 (7.7%)	9 (11.1%)	4 (4.6%)	0.11^c^
- No	155 (92.3%)	72 (88.9%)	83 (95.4%)	
**CD4 count (cells/μl)**^e^, **median (IQR); n = 89**	102 (49–182)	115 (59–209)	86 (44–136)	0.05^b^
**CD4 count (cells/μl), n (%)**				
- ≤100	44 (49.4%)	19 (44.2%)	25 (54.4%)	0.23^a^
- 101–199	27 (30.3%)	12 (27.9%)	15 (32.6%)	
- 200–349	15 (16.9%)	9 (20.9%)	6 (13.0%)	
- ≥350	3 (3.4%)	3 (7.0%)	0 (0.0%)	
**WHO stage, n (%); n = 138**				
- I/II/III	32 (23.2%)	13 (22.4%)	19 (23.8%)	0.85^c^
- IV	106 (76.8%)	45 (77.6%)	61 (76.3%)	
**Advanced HIV**^f^; **n = 151**				
- Yes	111 (73.5%)	48 (69.6%)	63 (76.8%)	0.31^c^
- No	40 (26.5%)	21 (30.4%)	19 (23.2%)	
**ART regimen**^g^, **n (%); n = 152**				
- Tenofovir based regimen	90 (59.2%)	48 (71.6%)	42 (49.4%)	<0.001^c^
- Non-tenofovir based regimen	45 (29.6%)	7 (10.5%)	38 (44.7%)	
- None	17 (11.2%)	12 (17.9%)	5 (5.9%)	
**ART initiated before VL episode, n (%); n = 165**				
- Yes^h^	106 (64.2%)	33 (42.3%)	73 (83.9%)	<0.001^c^
- No	59 (35.8%)	45 (57.7%)	14 (16.1%)	
**Parasite grade, median (IQR); n = 137**	5 (3–6)	4 (1–6)	5 (4–6)	0.003^b^
**Parasite grade, n (%)**^i^; **n = 170**				
- <6+	82 (48.2%)	36 (43.9%)	46 (52.3%)	<0.001^c^
- 6+	55 (32.4%)	15 (18.3%)	40 (45.5%)	
- Not done: serological/clinical diagnosis	33 (19.4%)	31 (37.8%)	2 (2.3%)	
**Cured after initial treatment, n (%); n = 145**				
- Parasitological cure	80 (55.2%)	28 (43.1%)	52 (65.0%)	0.008^c^
- Clinical cure	65 (44.8%)	37 (56.9%)	28 (35.0%)	

Abbreviations: ART, antiretroviral therapy; IQR, interquartile range; VL, visceral leishmaniasis.

^a^ Fisher’s exact test.

^b^ Two-sample Wilcoxon rank-sum (Mann-Whitney) test.

^c^ Chi-squared test.

^d^ Defined according to MSF guidelines as follows: [State of collapse (unable to sit up unaided and cannot drink unaided); severely weak (cannot walk 5 meters without assistance); other types of weakness were classified as “other”].

^e^ CD4 count result is <6 months from VL treatment initiation.

^f^ WHO stage IV or CD4 <50 cells/μL.

^g^ Stavudine, lamivudine and nevirapine; zidovudine, lamivudine and efavirenz; tenofovir, lamivudine and efavirenz; zidovudine, lamivudine and nevirapine; stavudine, lamivudine and efavirenz.

^h^ Of the 106 patients that started ART before the VL episode: 51 started tenofovir based regimen, 45 started non-tenofovir based regimen, and in 10 patients the ART regimen was missing. The overall results of “ART initiated before VL episode (in ART categories)” by “VL treatment history” are similar to those presented.

^i^132 (76.3%) spleen aspirates, 4 (2.3%) bone marrow aspirates, 1 lymph node aspirate (0.6%), 33 (19.1%) no parasitological test done (31 were primary VL), and 3 (1.7%) data missing. Overall– 90 (52.0%) parasitological diagnosis, 74 (42.8%) serological diagnosis, 6 (3.5%) clinical diagnosis (all were relapse VL) and 3 (1.7%) data missing.

Compared to relapse VL patients, a higher proportion of primary VL patients were aged 18–40 years (88.0% *vs*. 75.6%), had been ill for ≥2 months (46.2% *vs*. 27.9%) and had not started ART prior to the VL episode (57.7% *vs*. 16.1%). Lastly, primary VL patients had a lower parasite load at admission (median +4 *vs*. +5) and a lower proportion had cure confirmed by a parasitological test (43.1% *vs*. 65.0%) (Table 1).

### Initial treatment outcome

The outcomes were: cured, 145/173 (83.8%; 95% CI, 77.6–88.6); died, 22/173 (12.7%; 95% CI, 8.5–18.5) and parasitological failure, 6/173 (3.5%; 95% CI, 1.6–7.4). The outcome by VL treatment history was significantly different as shown in Table 2.

**Table 2:** Initial treatment outcomes of visceral leishmaniasis and HIV co-infected patients treated with a combination of liposomal amphotericin B (AmBisome) and miltefosine by MSF in Ethiopia from January 2011 to August 2014, by visceral leishmaniasis treatment history (N = 173).

Initial treatment outcome	Total, n/N (%)	Primary visceral leishmaniasis, n/N (%)	Relapse visceral leishmaniasis, n/N (%)	*P*
	95% confidence interval	95% confidence interval	95% confidence interval	
Cure	145/173 (83.8)	65/83 (78.3)	80/90 (88.9)	0.003^a^
	(77.6–88.6)	68.3–85.8	80.7–93.9	
Death	22/173 (12.7)	17/83 (20.5)	5/90 (5.6)	
	(8.5–18.5)	13.2–30.4	2.4–12.4	
Parasitological failure	6/173 (3.5)	1/83 (1.2)	5/90 (5.6)	
	(1.6–7.4)	0.2–6.5	2.4–12.4	

^a^ Fisher’s exact test.

Of the 6 patients with initial parasitological failure (Table 2), 1 was retreated with AmBisome and miltefosine combination, 2 with AmBisome alone and 3 with SSG based regimen. One of the patients retreated with SSG based regimen died, all the rest were cured. The treatment outcomes at discharge were: cured, 150/173 (86.7%); died, 23/173 (13.3%) and no parasitological failure.

### Predictors of initial parasitological failure

Tuberculosis co-infection at VL diagnosis was predictive of initial parasitological failure (adjusted odds ratio (aOR), 8.14; 95% CI, 1.42–46.72; p = 0.02). There was a statistically non-significant association between high tissue parasite load (parasite grade 6+) at VL diagnosis and initial parasitological failure. In multivariable analysis, VL treatment history was not significantly associated with initial parasitological failure (Table 3).

**Table 3:** Predictors and odds ratios for initial parasitological failure in visceral leishmaniasis and HIV co-infected patients treated with a combination of liposomal amphotericin B (AmBisome) and miltefosine by MSF in Ethiopia from January 2011 to August 2014 (N = 151).

Predictors	n/N (%)	Crude OR (95% CI)	*P*	Adjusted OR (95% CI)	*P*
**Age (years)**					
- 18–40	4/128 (3.1)	1.0	0.23^a^	–	–
- >40	2/23 (8.7)	2.95 (0.51–17.15)		–	
**Spleen size ≥11 cm**					
- No	5/127 (3.9)	1.0	1.00^a^	–	–
- Yes	1/21 (4.8)	1.22 (0.14–11.00)		–	
**Body mass index <16 kg/m**^**2**^					
- No	3/88 (3.4)	1.0	1.00^a^	–	–
- Yes	2/57 (3.5)	1.03 (0.17–6.37)		–	
**Tuberculosis**					
- No	2/116 (1.7)	1.0	0.02^a^	1.0	0.02
- Yes	4/32 (12.5)	8.14 (1.42–46.72)		8.14 (1.42–46.72)	
**Primary VL**					
- No	5/85 (5.9)	1.0	0.23^a^	–	–
- Yes	1/66 (1.5)	0.25 (0.03–2.16)		–	
**Advanced HIV**^b^					
- No^c^	0/34 (0.0)	1.0	0.33^a^	–	–
- Yes	5/98 (5.1)	–		–	
**Parasite grade**					
- <6+	1/74 (1.4)	1.0	0.04^a^	–	–
- 6+	5/50 (10.0)	8.11 (0.92–71.68)		–	
- Serological/clinical diagnosis^c^	0/24 (0.0)	–		–	
**ART initiated before VL episode**					
- Yes^d^	6/95 (6.3)	1.0	0.09^a^	–	–
- No^c^	0/50 (0.0)	–		–	

Abbreviations: ART, antiretroviral therapy; CI, confidence interval; OR, odds ratio; VL, visceral leishmaniasis.

^a^ Fisher’s exact test.

^b^ WHO stage IV or CD4 <50 cells/μL.

^c^ No patient with parasitological failure in these subgroups.

^d^ Of the 6 patients with parasitological failure out of the 95 patients that started ART before the VL episode: 3/44 started tenofovir based regimen, 3/41 started non-tenofovir based regimen, and in 10 patients the ART regimen was missing. The prediction of parasitological failure by the variable “ART initiated before VL episode (in ART categories)” are similar to those presented.

### Predictors of death

Independent predictors of death were age >40 years (aOR, 5.10; 95% CI, 1.50–17.44; p = 0.009), hemoglobin ≤6.5 g/dL (aOR, 5.20; 95% CI, 1.83–14.79; p = 0.002) and primary VL (aOR, 8.33; 95% CI, 2.27–30.63; p = 0.001) as shown in Table 4.

**Table 4:** Predictors and odds ratios for death in visceral leishmaniasis and HIV co-infected patients treated with a combination of liposomal amphotericin B (AmBisome) and miltefosine by MSF in Ethiopia from January 2011 to August 2014 (N = 173).

Predictors	n/N (%)	Crude OR (95% CI)	*P*	Adjusted OR (95% CI)	*P*
**Age (years)**					
- 18–40	13/141 (9.2)	1.0	0.007^a^	1.0	0.009
- >40	9/32 (28.1)	3.85 (1.48–10.05)		5.10 (1.50–17.44)	
**Spleen size ≥11 cm**					
- No	15/142 (10.6)	1.0	0.06^a^	–	–
- Yes	7/28 (25.0)	2.82 (1.03–7.74)		–	
**Hemoglobin ≤6.5 g/dL**					
- No	9/128 (7.0)	1.0	<0.001^b^	1.0	0.002
- Yes	12/40 (30.0)	5.67 (2.18–14.76)		5.20 (1.83–14.79)	
**Body mass index <16 kg/m**^**2**^					
- No	8/96 (8.3)	1.0	0.12^b^	–	–
- Yes	11/68 (16.2)	2.12 (0.80–5.60)		–	
**Tuberculosis**					
- No	15/131 (11.5)	1.0	0.29^b^	–	–
- Yes	7/39 (18.0)	1.70 (0.64–4.50)		–	
**Primary VL**					
- No	5/90 (5.6)	1.0	0.003^b^	1.0	0.001
- Yes	17/83 (20.5)	4.38 (1.54–12.48)		8.33 (2.27–30.63)	
**Duration of illness ≥2 months**					
- No	11/104 (10.6)	1.0	0.40^b^	–	–
- Yes	9/60 (15.0)	1.50 (0.58–3.84)		–	
**Bleeding**					
- No	19/162 (11.7)	1.0	0.12^a^	–	–
- Yes	2/5 (40.0)	5.02 (0.79–31.98)		–	
**Jaundice**					
- No	21/164 (12.8)	1.0	0.51^a^	–	–
- Yes	1/5 (20.0)	1.70 (0.18–15.97)		–	
**Weakness (severe/collapse)**^c^					
- No	19/143 (13.3)	1.0	1.00^a^	–	–
- Yes	3/27 (11.1)	0.82 (0.22–2.97)		–	
**Edema and/or ascites**					
- No	18/155 (11.6)	1.0	0.07^a^	–	–
- Yes	4/13 (30.8)	3.38 (0.94–12.12)		–	
**Advanced HIV**^d^					
- No	6/40 (15.0)	1.0	0.59^b^	–	–
- Yes	13/111 (11.7)	0.75 (0.26–2.13)		–	
**ART initiated before VL episode**					
- Yes^e^	11/106 (10.4)	1.0	0.36^b^	–	–
- No	9/59 (15.3)	1.55 (0.60–4.00)		–	
**Parasite grade**					
- <6+	8/82 (9.8)	1.0	0.04^a^	–	–
- 6+	5/55 (9.1)	0.93 (0.29–2.99)		–	
- Not done (serological/clinical diagnosis)	9/33 (27.3)	3.47 (1.20–9.99)		–	

Abbreviations: ART, antiretroviral therapy; CI, confidence interval; OR, odds ratio; VL, visceral leishmaniasis.

^a^ Fisher’s exact test.

^b^ Chi-squared test.

^c^ Defined according to MSF guidelines as follows: [State of collapse (unable to sit up unaided and cannot drink unaided); severely weak (cannot walk 5 meters without assistance); other types of weakness were classified as “other”].

^d^ WHO stage IV or CD4 <50 cells/μL.

^e^ Of the 11 dead patients out of the 106 patients that started ART before the VL episode: 7/51 started tenofovir based regimen, 4/45 started non-tenofovir based regimen, and in 10 patients the ART regimen was missing. The prediction of death by the variable “ART initiated before VL episode (in ART categories)” are similar to those presented.

### Sensitivity analysis

The initial treatment outcomes were similar to those from the main analysis as shown in S2 Table. As also reported in the main analysis, tuberculosis co-infection at VL diagnosis was predictive of initial parasitological failure. Additionally, BMI<16 kg/m^2^ (severe malnutrition) was also predictive of initial parasitological failure as shown in S3 Table. The independent predictors of death were similar to those from the main analysis as shown in S4 Table.

## Discussion

VL-HIV co-infected patients are confronted with high (initial) parasitological failure rates and high relapse rates [7,8]. While we recently identified pentamidine secondary prophylaxis as a promising option to reduce the relapse rates [9,11], achieving parasitological cure has been challenging. In this study, we determined the initial effectiveness (cure, death and parasitological failure rates) of a combination regimen of AmBisome and miltefosine for treatment of VL in HIV co-infected patients in Ethiopia. The initial cure rate was 83.8%, death rate 12.7% and parasitological failure rate 3.5%. Tuberculosis co-infection at VL diagnosis was predictive of initial parasitological failure. Age >40 years, hemoglobin level ≤6.5 g/dL and primary VL were predictive of death.

Although it remains difficult to compare historical cohorts, the initial treatment outcomes with combination therapy compared with those for AmBisome monotherapy—the previous first line treatment at the MSF treatment site [24], are as follows: parasitological failure rates were significantly lower (3.5% *vs*. 32.8%; p<0.001), cure rates were significantly higher (83.8% *vs*. 60.4%; p<0.001), and death rates were non-significantly higher (12.7% *vs*. 6.8%; p = 0.05). None of the deaths are considered treatment-related. In the present study, we had a higher admission rate of late stage VL patients that were referred from other hospitals as compared to the previous AmBisome monotherapy study. We have recently shown that besides HIV serostatus, other important predictors of death were: age >40 years, hemoglobin ≤6.5 g/dL, bleeding, jaundice, edema, ascites and tuberculosis [35]. We also found that in the presence of major predictors of death, the predictive effect of treatment on outcome may be minimal [35,36]. If we are to consider initial parasitological failure and death as overall initial failure (assuming that patients who died also had parasitological failure), then the overall initial failure rate with the combination regimen were also significantly lower than with AmBisome monotherapy (16.2% *vs*. 39.6%; p<0.001) [24].

In contrast to pentavalent antimonials that cause severe adverse events resulting in high case fatality rates [7,12–18], AmBisome and miltefosine have been shown to be safe [7,15,20,21,24,26]. Therefore the higher case fatality rates reported here are more likely related to the patients clinical conditions (late stage VL patients), than due to AmBisome and miltefosine toxicity [7,15,20,21,24,26]. Combination treatment may increase treatment efficacy and tolerance, reduce treatment duration and cost, and limit the emergence of drug resistance [23,25]. VL combination therapies have been successfully used and implemented in HIV-negative patients [37,38]. *In vitro* studies have demonstrated synergy between liposomal amphotericin B and miltefosine [39]. A combination of synergistic treatment regimens with different modes of action and mechanisms to develop resistance can also delay the emergence of drug-resistance [23]. AmBisome and miltefosine combination therapy was safe and effective in HIV-negative patients [38]. For co-infected patients, an Indian retrospective study on AmBisome and miltefosine combination therapy showed it was safe and effective, however, initial treatment outcomes were not reported [40].

Thanks to an agreement between Gilead and WHO, on a donation programme, WHO is providing AmBisome for treatment of visceral leishmaniasis for free to low income countries in East Africa and South Asia. This donation programme started in 2012, and has been extended in 2017 for another five years, including middle income countries. This access to free AmBisome will enhance affordability of implementing AmBisome-based treatment regimens for VL-HIV [41–44]. Combining AmBisome and miltefosine may be crucial: with lower initial parasitological failure rates, fewer patients required retreatment and therefore treatment duration was shortened. This promotes patient compliance, reduces risk of adverse events, and patient and health facility costs [23]. AmBisome must be transported and stored at temperatures below 25° centigrade [26]. Miltefosine may be teratogenic, therefore it is contraindicated during pregnancy, women of reproductive age must use effective contraception during and for 3 months after treatment [21]. Miltefosine may also cause gastrointestinal symptoms (nausea and vomiting) [21]. In this study, there was no treatment discontinuation secondary to gastrointestinal symptoms. Good tolerance to miltefosine has also been reported in other studies [15,40]. Overall, similarly as in India, our findings are encouraging in terms of efficacy of combination therapy. Data from clinical trials are now needed to enhance the evidence base. Importantly, studies evaluating AmBisome and miltefosine combination therapy in HIV patients have been conducted in Ethiopia [45] and started in India [46]. The trial findings are to be published soon.

We found that tuberculosis co-infection at VL diagnosis was predictive of initial parasitological failure. This is similar to findings from a study conducted in Sudan [47]. Tuberculosis causes immunosuppression which may inhibit parasite clearance, resulting in parasitological failure [47]. In another Ethiopian study, high tissue parasite load at VL diagnosis was shown to predict parasitological failure [16]. Possibly in the presence of underlying immunosuppression, a high parasite load on admission might be more difficult to clear. In our study, we also found an association between high tissue parasite load at VL diagnosis and initial parasitological failure, however, it was not statistically significant. This finding could be explained by the few outcomes observed in this study—only 6 patients with initial parasitological failure. In sensitivity analysis, BMI<16 kg/m^2^ (severe malnutrition) was predictive of initial parasitological failure. As with tuberculosis, severe malnutrition causes immunosuppression which may inhibit parasite clearance, resulting in parasitological failure.

The predictors of death identified—age >40 years and hemoglobin level ≤6.5 g/dL—are similar to those reported from other studies [35,48,49]. Patients aged >40 years, may have underlying co-morbidities (e.g. cardiovascular diseases), lower immunity and/or severe VL disease [48,50–53], which increases their risk to die. Severe anemia may cause congestive heart failure [54]. In comparison with relapse VL patients, we found that primary VL patients were more likely to die. The exact reason for this is unknown. However, since VL-HIV coinfection is a severe illness [7], and the risk of VL relapse is high (26% at one year) [8], it is probable that relapse VL patients may be more likely to be aware of the dangers of VL than primary VL patients, they may present to the health center with an earlier stage of illness in comparison with primary VL patients that may arrive with more end stage illness.

Most of our patients are young adult males who get infected with leishmania while working in the agricultural fields within the VL endemic region. HIV infection is also more common in young adults than children. As shown in Table 1, the lowest age in this VL-HIV cohort was 18 years old. However, if we were to treat a younger (<18 years old) VL-HIV co-infected patient cohort with this combination regimen, probably those <5 years old would have high case fatality rates, that would be comparable to those of patients aged >40 years in this cohort. This is because several studies have shown that younger HIV negative VL patients have higher risk of death [49,51,55]. AmBisome and miltefosine would still be the treatment of choice, however, an allometric dosing table for miltefosine should be used in children, as it might improve treatment outcomes [56].

There are some limitations to this study. Diagnosis and cure were not systematically confirmed by parasitological tests. Fifty-seven (88.0%) of the patients whose cure was assessed clinically had non or barely palpable spleen at the end-of-treatment, inhibiting performing a spleen aspirate, they declined having a bone marrow aspirate because the procedure is painful and they didn’t have palpable lymph nodes. This occurs commonly in settings without non-invasive investigations to assess VL cure. We acknowledge that this could likely lead to some degree of underestimation of the failure rates. However, it is important to note that in this cohort of patients, the more ill patients at admission (for instance with tuberculosis co-infection) and those more likely to fail (*e*.*g*. patients with a history of VL) were more likely to get a parasitological test for confirmation of cure at the end of treatment. Furthermore, in a recent study from this setting, we found no difference in long term outcomes (relapse or death) among patients with treatment outcome at discharge of parasitological cure versus those with clinical cure [8]. In a worst-case scenario, assuming similar failure rates for those with clinical cure compared to those undergoing tissue aspiration, the overall failure rates would still only be 6.4%, clearly better as what has been reported with miltefosine and AmBisome monotherapy. While longer patient follow-up to report on the relapse rates would have been of interest, this was not done as some patients were included in the pentamidine secondary prophylaxis trial, which has been published recently [11]. Indeed, to prevent relapse, secondary prophylaxis is likely the most important intervention, and not the initial treatment [10]. Consequently, the focus of this paper was on the initial effectiveness of the combination regimen in achieving parasitological cure, which is a prerequisite before starting secondary prophylaxis. In this study, CD4 counts were missing for a significant proportion of patients. Working in a remote area with relatively limited capacity, we did not have the capacity to perform autopsies. However, basing on clinical experience, some of the underlying causes of death include: severe anemia, severe pneumonia, tuberculosis, hepatic failure and sepsis [35,57,58]. Also, as a retrospective study, we could only study predictors from the collected variables.

In conclusion, we determined the initial effectiveness of a combination regimen of AmBisome and miltefosine for treatment of VL in HIV co-infected patients in Ethiopia. Initial parasitological failure rates were very low with AmBisome and miltefosine combination therapy when compared with the initial parasitological failure rates with either drug administered as monotherapy [15,24]. Therefore, this combination regimen seems a suitable VL treatment option in HIV patients. These findings remain to be confirmed in clinical trials. After achieving initial cure, those at high risk of VL relapse should be initiated on secondary prophylaxis.

## Supporting information

S1 Table Demographic and clinical characteristics of patients with visceral leishmaniasis and HIV co-infection that were excluded or included in the main (per-protocol) analysis (N = 227). (DOCX)

S2 Table Initial treatment outcomes (cure, death and parasitological failure—includes defaulters/transfer-outs), by visceral leishmaniasis treatment history (N = 182). (DOCX)

S3 Table Predictors and odds ratios for initial parasitological failure (includes defaulters/transfer-outs) in visceral leishmaniasis and HIV co-infected patients treated with a combination of liposomal amphotericin B (AmBisome) and miltefosine (N = 160). (DOCX)

S4 Table Predictors and odds ratios for death versus staying alive [cure and parasitological failure—includes defaulters/transfer-outs] in visceral leishmaniasis and HIV co-infected patients treated with a combination of liposomal amphotericin B (AmBisome) and miltefosine (N = 182). (DOCX)

S1 Checklist STROBE checklist. (DOC)
