# Supplementary material for: The initial effectiveness of liposomal amphotericin B (AmBisome) and miltefosine combination for treatment of visceral leishmaniasis in HIV co-infected patients in Ethiopia: A retrospective cohort study
Source: PLoS Negl Trop Dis. 2018 May 25;12(5):e0006527. doi: 10.1371/journal.pntd.0006527 (PMC5991765; doi:10.1371/journal.pntd.0006527)
Supplement: S1 Table — (DOCX) [file pntd.0006527.s001.docx]

**S1 Table. Demographic and clinical characteristics of patients with visceral leishmaniasis and HIV co-infection that were excluded or included in the main (per-protocol) analysis (N=227)**

| **Characteristic** | **Excluded (N=54)** | **Included (N=173)** | ***P*** |
| --- | --- | --- | --- |
| **Sex, n (%)** |  |  |  |
| - Male | 53 (98.2%) | 170 (98.3%) | 1.00^a^ |
| - Female | 1 (1.9%) | 3 (1.7%) |  |
| **Age (years), median (IQR)** | 32.0 (30.0−39.0) | 32.0 (28.0−39.0) | 0.32^b^ |
| **Age (years), n (%)** |  |  |  |
| - 18−40 | 45 (83.3%) | 141 (81.5%) | 0.76^c^ |
| - >40 | 9 (16.7%) | 32 (18.5%) |  |
| **Residential status, n (%); n=225** |  |  |  |
| - Migrant worker | 21 (38.9%) | 70 (40.9%) | 0.79^c^ |
| - Resident | 33 (61.1%) | 101 (59.1%) |  |
| **Spleen size (cm), median (IQR); n=223** | 6.0 (4.0−10.0) | 6.0 (4.0−10.0) | 0.84^b^ |
| **Spleen size >11 cm, n (%)** |  |  |  |
| - Yes | 10 (18.9%) | 28 (16.5%) | 0.69^c^ |
| - No | 43 (81.1%) | 142 (83.5%) |  |
| **Hemoglobin level (g/dL), median (IQR); n=221** | 7.2 (6.5−9.1) | 8.2 (6.7−9.2) | 0.15^b^ |
| **Hemoglobin level <6.5 g/dL, n (%)** |  |  |  |
| - Yes | 15 (28.3%) | 40 (23.8%) | 0.51^c^ |
| - No | 38 (71.7%) | 128 (76.2%) |  |
| **Body mass index (kg/m^2^), median (IQR); n=214** | 16 (15−17) | 16 (15−18) | 0.24^b^ |
| **Body mass index** <16 **Kg/m^2^, n (%)** |  |  |  |
| - Yes | 25 (50.0%) | 68 (41.5%) | 0.29^c^ |
| - No | 25 (50.0%) | 96 (58.5%) |  |
| **Tuberculosis, n (%); n=223** |  |  |  |
| - Yes | 11 (20.8%) | 39 (22.9%) | 0.74^c^ |
| - No | 42 (79.3%) | 131 (77.1%) |  |
| **Jaundice, n (%); n=223** |  |  |  |
| - Yes | 1 (1.9%) | 5 (3.0%) | 1.00^a^ |
| - No | 53 (98.2%) | 164 (97.0%) |  |
| **Duration of illness (months), median (IQR); n=217** | 1.0 (1.0−2.0) | 1.0 (1.0−2.0) | 0.69^b^ |
| **Duration of illness >2** **months, n (%)** |  |  |  |
| - Yes | 18 (34.0%) | 60 (36.6%) | 0.73^c^ |
| - No | 35 (66.0%) | 104 (63.4%) |  |
| **Bleeding, n (%); n=217** |  |  |  |
| - Yes | 2 (4.0%) | 5 (3.0%) | 0.66^a^ |
| - No | 48 (96.0%) | 162 (97.0%) |  |
| **Weakness, n (%)^d^; n=224** |  |  |  |
| - Collapse | 0 (0.0%) | 1 (0.6%) | 0.18^a^ |
| - Severe | 14 (25.9%) | 26 (15.3%) |  |
| - Other | 40 (74.1%) | 143 (84.1%) |  |
| **Edema and/or ascites, n (%); n=221** |  |  |  |
| - Yes | 3 (5.7%) | 13 (7.7%) | 0.77^a^ |
| - No | 50 (94.3%) | 155 (92.3%) |  |
| **CD4 count (cells/µl)^e^, median (IQR); n=116** | 131 (49−300) | 102 (49−182) | 0.20^b^ |
| **CD4 count (cells/µl), n (%)** |  |  |  |
| - ≤100 | 10 (37.0%) | 44 (49.4%) | 0.18^a^ |
| - 101−199 | 8 (29.6%) | 27 (30.3%) |  |
| - 200−349 | 5 (18.5%) | 15 (16.9%) |  |
| - >350 | 4 (14.8%) | 3 (3.4%) |  |
| **WHO stage, n (%); n=171** |  |  |  |
| - I/II/III | 10 (30.3%) | 32 (23.2%) | 0.40^c^ |
| - IV | 23 (69.7%) | 106 (76.8%) |  |
| **Advanced HIV^f^; n=192** |  |  |  |
| - Yes | 27 (65.9%) | 111 (73.5%) | 0.33^c^ |
| - No | 14 (34.2%) | 40 (26.5%) |  |
| **ART regimen^g^, n (%); n=192** |  |  |  |
| - Tenofovir based regimen | 21 (52.5%) | 90 (59.2%) | 0.03^c^ |
| - Non-tenofovir based regimen | 8 (20.0%) | 45 (29.6%) |  |
| - None | 11 (27.5%) | 17 (11.2%) |  |
| **ART initiated before VL episode, n (%); n=211** |  |  |  |
| - Yes^h^ | 22 (47.8%) | 106 (64.2%) | 0.04^c^ |
| - No | 24 (52.2%) | 59 (35.8%) |  |
| **Parasite grade, median (IQR); n=161** | 4 (1−6) | 5 (3−6) | 0.38^b^ |
| **Parasite grade, n (%); n=215** |  |  |  |
| - <6+ | 14 (31.1%) | 82 (48.2%) | 0.001^c^ |
| - 6+ | 10 (22.2%) | 55 (32.4%) |  |
| - Not done: serological/clinical diagnosis | 21 (46.7%) | 33 (19.4%) |  |
| **VL treatment history, n (%)** |  |  |  |
| - Primary VL | 32 (59.3%) | 83 (48.0%) | 0.15^c^ |
| - Relapse VL | 22 (40.7%) | 90 (52.0%) |  |

Abbreviations: ART, antiretroviral therapy; IQR, interquartile range; VL, visceral leishmaniasis.

^a^ Fisher’s exact test.

^b^ Two-sample Wilcoxon rank-sum (Mann-Whitney) test.

^c^ Chi-squared test.

^d^ Defined according to MSF guidelines as follows: [State of collapse (unable to sit up unaided and cannot drink unaided); severely weak (cannot walk 5 meters without assistance); other types of weakness were classified as “other”].

^e^ CD4 count result is <6 months from VL treatment initiation.

^f^ WHO stage IV or CD4 <50 cells/μL.

^g^ Stavudine, lamivudine and nevirapine; zidovudine, lamivudine and efavirenz; tenofovir, lamivudine and and efavirenz; tenofovir, lamivudine and nevirapine; zidovudine, lamivudine and nevirapine; stavudine, lamivudine and efavirenz.

^h^ Of the 128 patients that started ART before the VL episode: 62 started tenofovir based regimen, 52 started non-tenofovir based regimen, and in 14 patients the ART regimen was missing. The overall results of “ART initiated before VL episode (in ART categories)” by “inclusion and exclusion status” are were not significantly different (*P*=0.07).
