# Supplementary material for: The initial effectiveness of liposomal amphotericin B (AmBisome) and miltefosine combination for treatment of visceral leishmaniasis in HIV co-infected patients in Ethiopia: A retrospective cohort study
Source: PLoS Negl Trop Dis. 2018 May 25;12(5):e0006527. doi: 10.1371/journal.pntd.0006527 (PMC5991765; doi:10.1371/journal.pntd.0006527)
Supplement: S2 Table — (DOCX) [file pntd.0006527.s002.docx]

**S2 Table. Initial treatment outcomes (cure, death and parasitological failure** − **includes defaulters/transfer-outs), by visceral leishmaniasis treatment history (N=182)**

| **Initial treatment outcome** | **Total, n/N (%)** | **Primary visceral leishmaniasis, n/N (%)** | **Relapse visceral leishmaniasis, n/N (%)** | ***P*** |
| --- | --- | --- | --- | --- |
|  | **95% confidence interval** | **95% confidence interval** | **95% confidence interval** |  |
| Cure | 145/182 (79.7) | 65/92 (70.7) | 80/90 (88.9) | 0.008^a^ |
|  | (73.2−84.9) | 60.7−79.0 | 80.7−93.9 |  |
| Death | 22/182 (12.1) | 17/92 (18.5) | 5/90 (5.6) |  |
|  | (8.1−17.6) | 11.9−27.6 | 2.4−12.4 |  |
| Parasitological failure^b^ | 15/182 (8.2) | 10/92 (10.9) | 5/90 (5.6) |  |
|  | (5.1−13.2) | 6.0−18.9 | 2.4−12.4 |  |

^a^ Chi-squared test.

^b^ Includes defaulters and transfer-outs.
