# Supplementary material for: The initial effectiveness of liposomal amphotericin B (AmBisome) and miltefosine combination for treatment of visceral leishmaniasis in HIV co-infected patients in Ethiopia: A retrospective cohort study
Source: PLoS Negl Trop Dis. 2018 May 25;12(5):e0006527. doi: 10.1371/journal.pntd.0006527 (PMC5991765; doi:10.1371/journal.pntd.0006527)
Supplement: S3 Table — (DOCX) [file pntd.0006527.s003.docx]

**S3 Table. Predictors and odds ratios for initial parasitological failure** (**includes defaulters/transfer-outs) in visceral leishmaniasis and HIV co-infected patients treated with a combination of liposomal amphotericin B (AmBisome) and miltefosine (N=160)**

| **Predictors** | **n/N (%)** | **Crude OR (95% CI)** | ***P*** | **Adjusted OR (95% CI)** | ***P*** |
| --- | --- | --- | --- | --- | --- |
| **Age (years)** |  |  |  |  |  |
| - 18−40 | 12/136 (8.8) | 1.0 | 0.70^a^ | − | − |
| - >40 | 3/24 (12.5) | 1.48 (0.38−5.68) |  | − |  |
| **Spleen size >11 cm** |  |  |  |  |  |
| - No | 12/134 (9.0) | 1.0 | 0.46^a^ | − | − |
| - Yes | 3/23 (13.0) | 1.53 (0.40−5.89) |  | − |  |
| **Body mass index <16 kg/m^2^** |  |  |  |  |  |
| - No | 4/89 (4.5) | 1.0 | 0.02^b^ | 1.0 | 0.04 |
| - Yes | 10/65 (15.4) | 3.86 (1.15−12.93) |  | 3.67 (1.04−12.92) |  |
| **Tuberculosis** |  |  |  |  |  |
| - No | 7/121 (5.8) | 1.0 | 0.02^a^ | 1.0 | 0.01 |
| - Yes | 7/35 (20.0) | 4.07 (1.32−12.56) |  | 4.78 (1.45−15.81) |  |
| **Primary VL** |  |  |  |  |  |
| - No | 5/85 (5.9) | 1.0 | 0.11^b^ | − | − |
| - Yes | 10/75 (13.3) | 2.46 (0.80−7.56) |  | − |  |
| **Advanced HIV^b^** |  |  |  |  |  |
| - No^c^ | 3/37 (8.1) | 1.0 | 1.00^a^ | − | − |
| - Yes | 9/102 (8.8) | 1.10 (0.28−4.29) |  | − |  |
| **Parasite grade** |  |  |  |  |  |
| - <6+ | 4/77 (5.2) | 1.0 | 0.17^a^ | − | − |
| - 6+ | 7/52 (13.5) | 2.84 (0.79−10.25) |  | − |  |
| - Serological/clinical diagnosis^c^ | 4/28 (14.3) | 3.04 (0.71−13.11) |  | − |  |
| **ART initiated before VL episode** |  |  |  |  |  |
| - Yes^d^ | 9/98 (9.2) | 1.0 | 1.00^a^ | − | − |
| - No^c^ | 4/54 (7.4) | 0.79 (0.23−2.70) |  | − |  |

Abbreviations: ART, antiretroviral therapy; CI, confidence interval; OR, odds ratio; VL, visceral leishmaniasis.

^a^ Fisher’s exact test.

^b^ Chi-squared test.

^c^ WHO stage IV or CD4 <50 cells/μL.

^d^ Of the 9 patients with parasitological failure (includes defaulters/transfer-outs) out of the 98 patients that started ART before the VL episode: 4/45 started tenofovir based regimen, 3/41 started non-tenofovir based regimen, and in 12 patients the ART regimen was missing (includes 2 with parasitological failure). The prediction of parasitological failure (includes defaulters/transfer-outs) by the variable “ART initiated before VL episode (in ART categories)” are similar to those presented.
