# Supplementary material for: The initial effectiveness of liposomal amphotericin B (AmBisome) and miltefosine combination for treatment of visceral leishmaniasis in HIV co-infected patients in Ethiopia: A retrospective cohort study
Source: PLoS Negl Trop Dis. 2018 May 25;12(5):e0006527. doi: 10.1371/journal.pntd.0006527 (PMC5991765; doi:10.1371/journal.pntd.0006527)
Supplement: S4 Table — (DOCX) [file pntd.0006527.s004.docx]

**S4 Table. Predictors and odds ratios for death versus staying alive [cure and parasitological failure** − **includes defaulters/transfer-outs] in visceral leishmaniasis and HIV co-infected patients treated with a combination of liposomal amphotericin B (AmBisome) and miltefosine (N=182)**

| **Predictors** | **n/N (%)** | **Crude OR (95% CI)** | ***P*** | **Adjusted OR (95% CI)** | ***P*** |
| --- | --- | --- | --- | --- | --- |
| **Age (years)** |  |  |  |  |  |
| - 18−40 | 13/149 (8.7) | 1.0 | 0.007^a^ | 1.0 | 0.009 |
| - >40 | 9/33 (27.3) | 3.92 (1.51−10.19) |  | 4.77 (1.47−15.43) |  |
| **Spleen size >11 cm** |  |  |  |  |  |
| - No | 15/149 (10.1) | 1.0 | 0.06^a^ | − | − |
| - Yes | 7/30 (23.3) | 2.72 (1.00−7.39) |  | − |  |
| **Hemoglobin level <6.5 g/dL** |  |  |  |  |  |
| - No | 9/132 (6.8) | 1.0 | <0.001^b^ | 1.0 | 0.007 |
| - Yes | 12/45 (26.7) | 4.97 (1.93−12.80) |  | 4.04 (1.47−11.11) |  |
| **Body mass index <16 kg/m^2^** |  |  |  |  |  |
| - No | 8/97 (8.3) | 1.0 | 0.19^b^ | − | − |
| - Yes | 11/76 (14.5) | 1.88 (0.72−4.94) |  | − |  |
| **Tuberculosis** |  |  |  |  |  |
| - No | 15/136 (11.0) | 1.0 | 0.33^b^ | − | − |
| - Yes | 7/42 (16.7) | 1.61 (0.61−4.27) |  | − |  |
| **Primary VL** |  |  |  |  |  |
| - No | 5/90 (5.6) | 1.0 | 0.007^b^ | 1.0 | 0.004 |
| - Yes | 17/92 (18.5) | 3.85 (1.36−10.95) |  | 6.43 (1.82−22.64) |  |
| **Duration of illness >2** **months** |  |  |  |  |  |
| - No | 11/107 (10.3) | 1.0 | 0.50^b^ | − | − |
| - Yes | 9/66 (13.6) | 1.38 (0.54−3.53) |  | − |  |
| **Bleeding** |  |  |  |  |  |
| - No | 19/168 (11.3) | 1.0 | 0.16^a^ | − | − |
| - Yes | 2/6 (33.3) | 3.92 (0.67−22.87) |  | − |  |
| **Jaundice** |  |  |  |  |  |
| - No | 21/173 (12.1) | 1.0 | 0.49^a^ | − | − |
| - Yes | 1/5 (20.0) | 1.81 (0.19−16.97) |  | − |  |
| **Weakness (severe/collapse)^c^** |  |  |  |  |  |
| - No | 19/148 (12.8) | 1.0 | 0.77^a^ | − | − |
| - Yes | 3/31 (9.7) | 0.73 (0.20−2.63) |  | − |  |
| **Edema and/or ascites** |  |  |  |  |  |
| - No | 18/164 (11.0) | 1.0 | 0.06^a^ | − | − |
| - Yes | 4/13 (30.8) | 3.60 (1.00−12.91) |  | − |  |
| **Advanced HIV^d^** |  |  |  |  |  |
| - No | 6/43 (14.0) | 1.0 | 0.65^b^ | − | − |
| - Yes | 13/115 (11.3) | 0.79 (0.28−2.22) |  | − |  |
| **ART initiated before VL episode** |  |  |  |  |  |
| - Yes^e^ | 11/109 (10.1) | 1.0 | 0.41^b^ | − | − |
| - No | 9/63 (14.3) | 1.48 (0.58−3.81) |  | − |  |
| **Parasite grade** |  |  |  |  |  |
| - <6+ | 8/85 (9.4) | 1.0 | 0.06^a^ | − | − |
| - 6+ | 5/57 (8.8) | 0.93 (0.29−2.99) |  | − |  |
| - Serological/clinical diagnosis | 9/37 (24.3) | 3.09 (1.09−8.81) |  | − |  |

Abbreviations: ART, antiretroviral therapy; CI, confidence interval; OR, odds ratio; VL, visceral leishmaniasis.

^a^ Fisher’s exact test.

^b^ Chi-squared test.

^c^ Defined according to MSF guidelines as follows: [State of collapse (unable to sit up unaided and cannot drink unaided); severely weak (cannot walk 5 meters without assistance); other types of weakness were classified as “other”].

^d^ WHO stage IV or CD4 <50 cells/μL. ^e^ Of the 11 dead patients out of the 109 patients that started ART before the VL episode: 7/52 started tenofovir based regimen, 4/45 started non-tenofovir based regimen, and in 12 patients the ART regimen was missing. The prediction of death by the variable “ART initiated before VL episode (in ART categories)” are similar to those presented.
